# Diencephalic progenitors contribute to the posterior septum through rostral migration along the hippocampal axonal pathway

**DOI:** 10.1038/s41598-018-30020-9

**Published:** 2018-08-06

**Authors:** Keisuke Watanabe, Koichiro Irie, Carina Hanashima, Hirohide Takebayashi, Noboru Sato

**Affiliations:** 1 Division of Gross Anatomy and Morphogenesis, Graduate School of Medical and Dental Sciences, Niigata University, Niigata, 951-8510 Japan; 2 Faculty of Education and Integrated Arts and Sciences, Waseda University, Shinjuku-ku, Tokyo 162-8480 Japan; 3 Graduate School of Advanced Science and Engineering, Waseda University, Shinjuku-ku, Tokyo 162-8480 Japan; 4 Division of Neurobiology and Anatomy, Graduate School of Medical and Dental Sciences, Niigata University, Niigata, 951-8510 Japan

## Abstract

Septal nuclei are telencephalic structures associated with a variety of brain functions as part of the limbic system. The two posterior septal nuclei, the triangular septal nucleus (TS) and the bed nuclei of the anterior commissure (BAC), are involved in fear and anxiety through their projections to the medial habenular nucleus. However, the development of both the TS and BAC remains unclear. Here, we found a novel caudal origin and putative migratory stream of mouse posterior septal neurons arising from the thalamic eminence (TE), a transient developmental structure at the rostral end of the rodent diencephalon. TE-derived cells, which have glutamatergic identity, migrated rostrally and entered the telencephalic territory by passing beneath the third ventricle. Subsequently, they turned dorsally toward the posterior septum. We also observed that TS and BAC neurons in the postnatal septum were labeled with GFP by in utero electroporation into the TE, suggesting a shared origin. Furthermore, TE-derived septal neurons migrated along the fornix, an efferent pathway from the hippocampus. These results demonstrate that posterior septal neurons have a distinct extratelencephalic origin from other septal nuclei. This heterogeneous origin may contribute to neuronal diversity of the septal nuclear complex.

## Introduction

**Figure 1:**
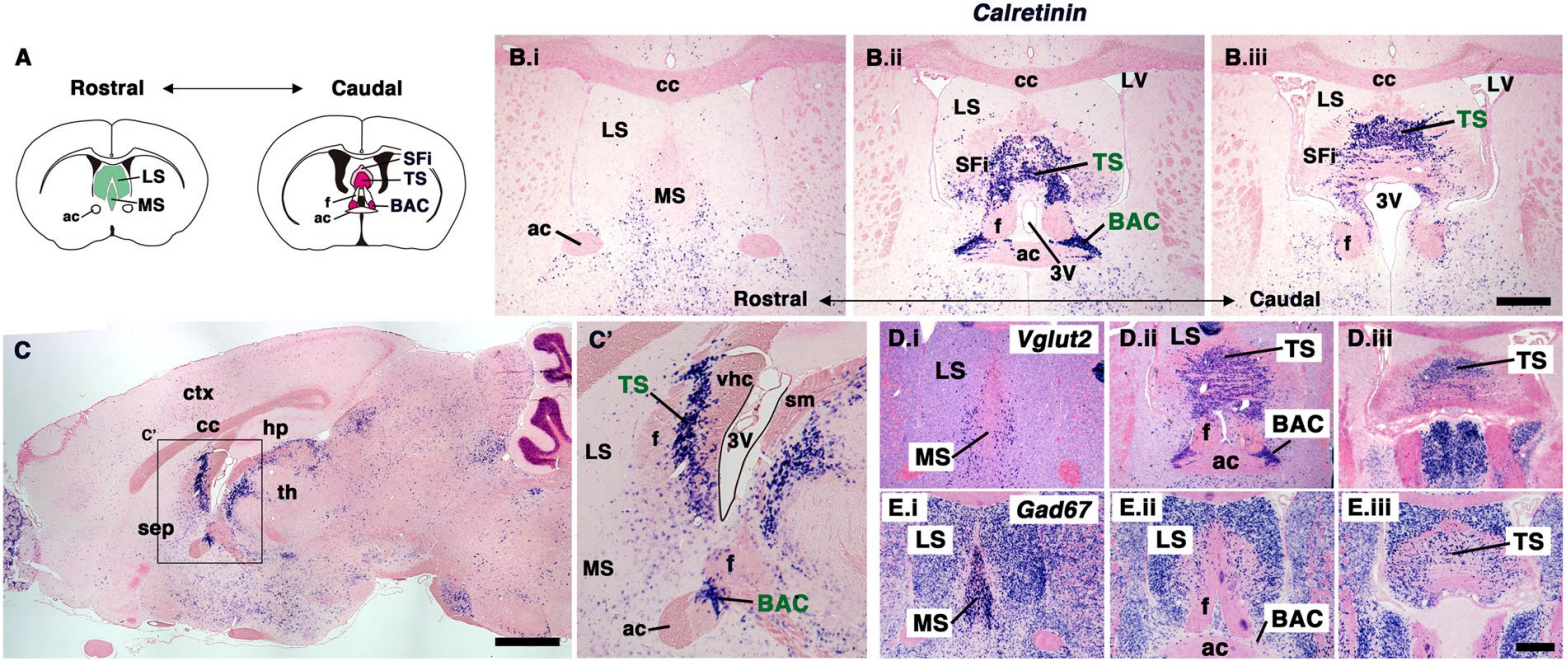
*Calretinin*-positive TS and BAC neurons in the posterior septal nuclei. (**A**) Schematic diagram showing the rostral to caudal septal nuclei. (**B**,**C**) Expression pattern of *Calretinin* (*CalR*) visualized by ISH in the adult mouse brain. B.i-B.iii show rostral to caudal coronal sections of the mouse brain. C shows a sagittal plane; the boxed area is magnified in C’. *CalR* was highly expressed in both the TS and BAC. (**D**,**E**) Expression of *Vglut2* (**D**) and *Gad67* (**E**) in the rostral to caudal septal nuclei. Both TS and BAC neurons expressed the glutamatergic neuron marker, *Vglut2*. ac, anterior commissure; BAC, bed nuclei of the anterior commissure; cc, corpus callosum; ctx, cerebral cortex; f, fornix; hp, hippocampus; LS, lateral septal nucleus; LV, lateral ventricle; MS, medial septal nucleus; sep, septal nuclei; SFi, septofimbrial nucleus; sm, stria medullaris; th, thalamus; TS, triangular septal nucleus; vhc, ventral hippocampal commissure; 3 V, third ventricle. Scale bars: 500 µm in B,D,E and 1 mm in C.

The septum (septal nuclei), considered part of the limbic system, is composed of the subcortical telencephalic structures that lie close to the midline and anterior to the lamina terminalis. The septum is broadly divided into four regions based on their anatomical location: the lateral, medial, posterior, and ventral groups^1^. The septal regions with the most well-studied function and connectivity are the lateral septal (LS) and medial septal (MS) nuclei^1,2^ (Fig. 1A). In contrast, the triangular septal nucleus (TS) and the bed nuclei of the anterior commissure (BAC) are categorized as posterior nuclei. TS and BAC neurons, which project to the medial habenula nuclei (MHb) through the stria medullaris, are associated with fear and anxiety^3,4^. Recently, it was shown that ablation of TS and BAC neurons resulted in impaired anxiety-related and fear-directed behaviors, respectively, suggesting their importance in these emotional behaviors^5^. In addition, septal functions have been associated with Alzheimer’s disease and schizophrenia^2,6,7^. Thus, the characterization of septal development is important for understanding its functional organization.

During development, neurons in the MS and LS are generated from local progenitors in the subpallium as well as from neighboring progenitor regions of the telencephalon, such as the medial ganglionic eminence (MGE), preoptic area (POA), and medial pallial-subpallial boundary (mPSB)^8,9^. Septal Nkx2-1 is required for the development of cholinergic neurons in the forebrain including the septum, and loss of *Nkx2-1* causes deficiencies in learning and memory^10^. However, the developmental origins of TS and BAC neurons remain unexplored.

During development of the telencephalon, specific types of glutamatergic excitatory and GABAergic inhibitory neurons have distinct origins^11,12^. For example, during corticogenesis, cortical GABAergic neurons originate from the embryonic subpallium followed by tangential migration to the cortex, whereas glutamatergic neurons are locally generated in the dorsal pallium. The MS and LS are rich in GABAergic neurons^1,13,14^, whereas the TS is enriched with glutamatergic neurons^15,16^. Interestingly, both TS and BAC neurons express high levels of Vglut2, which is mainly expressed in excitatory neurons of the diencephalic and brain stem regions^17^. These observations suggest that the posterior septal neurons are generated from an extra-telencephalic source, such as the neuroepithelium of the diencephalon.

In this study, we identified the thalamic eminence (TE; eminentia thalami [EmT]) as a novel caudal source of the posterior septal nuclei, the TS and BAC. The TE is described as a transient developmental structure located at the rostral end of the developing mammalian diencephalon, and as a forebrain hem system at the diencephalic-telencephalic boundary^18–21^. Cell migration from the diencephalon to the telencephalic regions has been implicated on the basis of gene expression patterns^19,22^. It was reported that the TE produces a subset of Cajal-Retzius cells, which control the radial migration of cortical neurons^23–25^. Furthermore, the posterior accessory olfactory bulb (pAOB) neurons also arise from the TE^25^. More recently, it was revealed that lot cells, which guide axons of the lateral olfactory tract, originate in the lateral part of the TE^26^. These studies indicate that TE-derived cells travel a long distance and differentiate into various neurons in diverse areas of the forebrain. Using both *in vivo* and *in vitro* studies, we identified a novel rostrodorsal migratory stream that emanates from the TE. TE-derived cells migrated rostrally beneath the third ventricle and, after entering the telencephalic regions, turned toward the prospective posterior septum. We also observed that they settled into both the TS and BAC during the postnatal stage. These septal nuclei comprise multiple neuronal subtypes and are interconnected with various parts of the brain, such as the hippocampus and hypothalamus^1,27^. Our findings indicate that the heterogeneous developmental origins of septal neurons may contribute to the neuronal diversity and functions of the septal nuclei.

## Results

### Distribution of *Calretinin*-positive neurons in the adult TS and BAC

To elucidate the developmental origins of neurons in the posterior septum, we first characterized the neuronal properties of the posterior septum in adult mouse brains. Calretinin (CalR), a calcium-binding protein, was reported to be expressed in almost all neurons of the posterior septum (the triangular septal nucleus [TS] and bed nuclei of the anterior commissure [BAC])^28^. We confirmed that *CalR* mRNA was highly expressed in both the TS and BAC, which are located at the midline of the subcortical regions and are bilaterally adjacent to the anterior commissure, respectively, at the posterior level of the septal nuclei (Fig. 1A,B). In addition, the septofimbrial nucleus (SFi), defined as the lateral group of the septum^1^, also showed strong expression of *CalR* (Fig. 1B). In sagittal sections of the adult brain, the TS was observed between two axonal tracts, the fornix and ventral hippocampal commissure (vhc), and the BAC was situated between the anterior commissure and fornix^29^ (Fig. 1C). This suggested that CalR may be suitable for tracing the development of TS and BAC neurons in embryonic as well as postnatal brains, since neuronal cells in the forebrain begin to express CalR from early developmental stages^22^.

As reported previously, both the TS and BAC are enriched with glutamatergic neurons, which were labeled with *Vesicular glutamate transporter 2* (*Vglut2*), whereas both the MS and LS are rich in GABAergic neurons, labeled with G*lutamic acid decarboxylase 67* (*Gad67*)^13–16^ (Fig. 1D,E). These observed differences in the neuronal composition between the posterior septum and other septal nuclei raised the possibility of their distinct origins.

### Developmental distribution of Calretinin-positive cells in the forebrain

**Figure 2:**
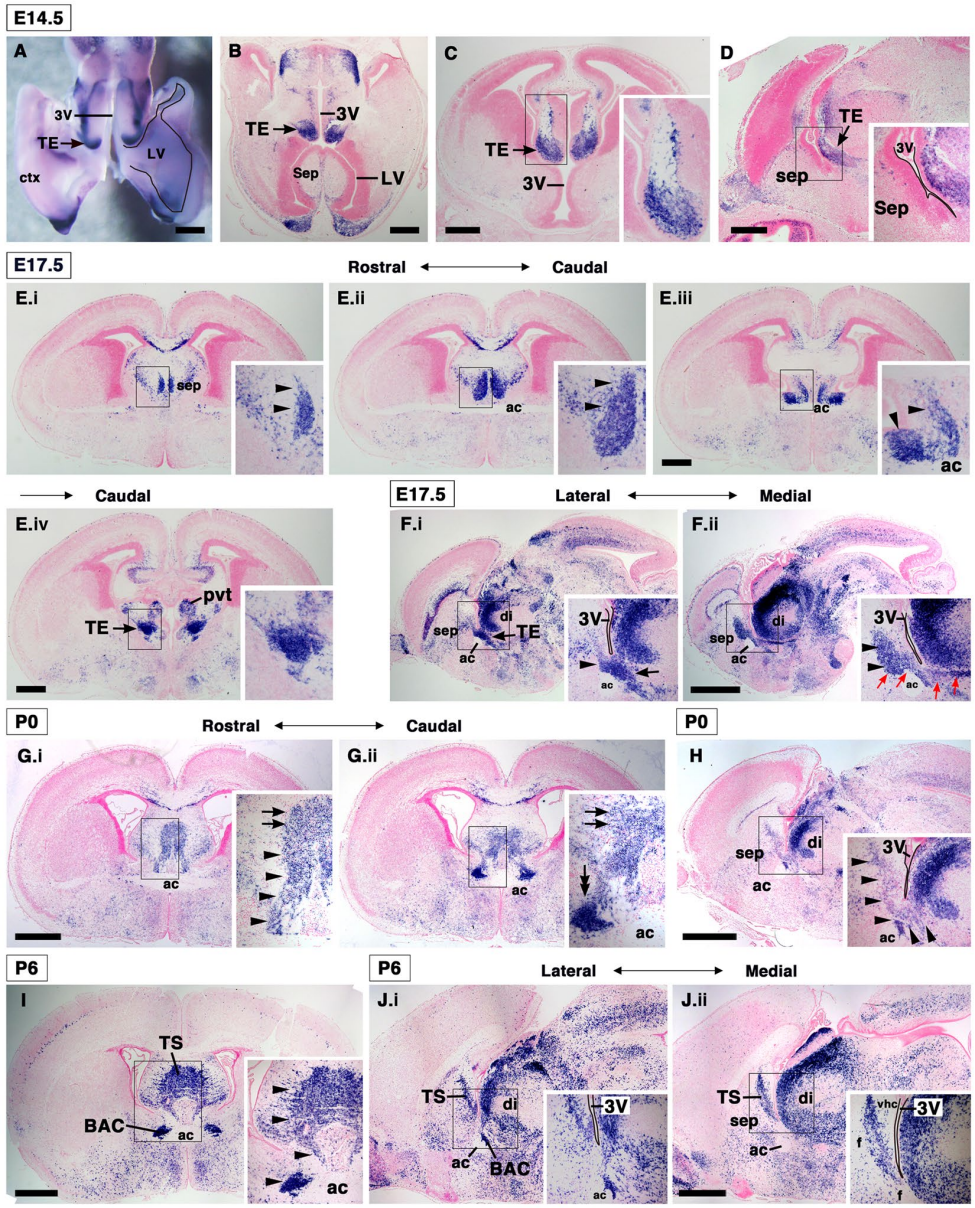
Expression pattern of *Calretinin* during development of the septal nuclei. Expression of *CalR* in the developing forebrain by ISH. (**A**–**D**) *CalR* expression at E14.5; (**A**) wholemount ISH, (**B**) horizontal section, (**C**) coronal section, and (**D**) sagittal section. The thalamic eminence (TE) showed a dense distribution of *CalR*-positive cells at E14.5. (**E**) Coronal sections from rostral (E.i) to caudal (E.iv) in the E17.5 forebrain. (**F**) E17.5 sagittal sections at two different levels. A stream of *CalR*-expressing cells from the TE to the prospective septal area was observed at this stage (arrowheads). (**G**) Coronal images from P0 mice. (**H**) P0 sagittal section. In the P0 prospective posterior septal region, the dorsoventral distribution of *CalR*-positive cells was still observed (arrowheads). (**I**) P6 coronal image. (**J**) Sagittal sections from P6 brains at two different levels. Both the TS and BAC were clearly distinguishable until this stage (arrowheads). Insets show a magnified view of the boxed areas. ac, anterior commissure; BAC, bed nuclei of the anterior commissure; ctx, cerebral cortex; di, diencephalon (thalamus); (**F**) fornix; hp, hippocampus; LV, lateral ventricle; pvt, paraventricular thalamic nucleus; sep, septal nuclei; TE, thalamic eminence; th, thalamus; TS, triangular septal nucleus; 3 V, third ventricle. Scale bars: 500 µm in A-E and 1 mm in F-J.

We next studied the embryonic origins of TS and BAC neurons by analyzing the expression patterns of *CalR* as a putative developmental marker. *CalR* is highly expressed in the thalamic eminence (TE; eminentia thalami [EmT]), which is a transient structure located at the rostral end of the diencephalon, from mouse embryonic day 11 (E11), and the expression in the TE is maintained through the late embryonic stage^21,22,30^. Consistent with this, at E14.5 a high level of *CalR* expression was observed in the TE^22^ (Fig. 2A–D). At the more rostral level at this stage, regions with a dense distribution of *CalR*-positive cells were restricted to the rostral septum, cortical hem, and olfactory bulb of the forebrain (Supplementary Fig. S1). Few *CalR*-positive cells were found in the prospective posterior septum (the region anterior to the third ventricle) at this stage (Fig. 2B,D, Supplementary Fig. S1). At E17.5, *CalR*-labeled TE cells were found close to the paraventricular nuclei of the thalamus (PVT) in caudal sections^22^ (Fig. 2E.iv). Interestingly, at a more rostral level, many *CalR*-positive cells were found close to the midline of the telencephalon (Fig. 2E.i,E.ii, arrowheads). These were bilaterally aligned along the dorsoventral axis and positioned dorsal to the anterior commissure (Fig. 2E.ii,E.iii). This cell distribution pattern was more prominent in sagittal sections of the E17.5 brain. In the sagittal plane, the TE was observed at the rostral end of the diencephalon^22^ (Fig. 2F.i, arrow). In more medial sections, many *CalR*-positive cells were accumulated in the developing septal region that is flanked caudally by the third ventricle (Fig. 2F.ii, arrowheads). Furthermore, cohorts of *CalR*-positive cells formed a caudal-to-rostral stream from the TE to the caudal portion of the emerging septal nuclei (Fig. 2F.ii, red arrows). This stream, which is equivalent to the dorsoventral distribution of *CalR*-positive cells observed in coronal sections, was not observed in E14.5 brains (Fig. 2D, Supplementary Fig. S1), suggesting the migration of *CalR*-positive cells from the TE. At P0, the dorsoventral pattern of *CalR*-positive cells was still observed in the developing septum (Fig. 2G.i,H, arrowheads), and putative *CalR*-positive TS neurons were found at the midline of the emerging septum (Fig. 2G.i,G.ii, arrows). Furthermore, densely packed *CalR*-positive cells were bilaterally found dorsal to the anterior commissure, where the BAC is located (Fig. 2G.ii, double-arrows). At this stage, the TE could not be distinguished^22^. At P6, the continuous stream of *CalR*-positive cells had largely disappeared (Fig. 2I,J). Consequently, two posterior septal nuclei, the TS and BAC, could be clearly recognized at this postnatal stage (Fig. 2I,J).

**Figure 3:**
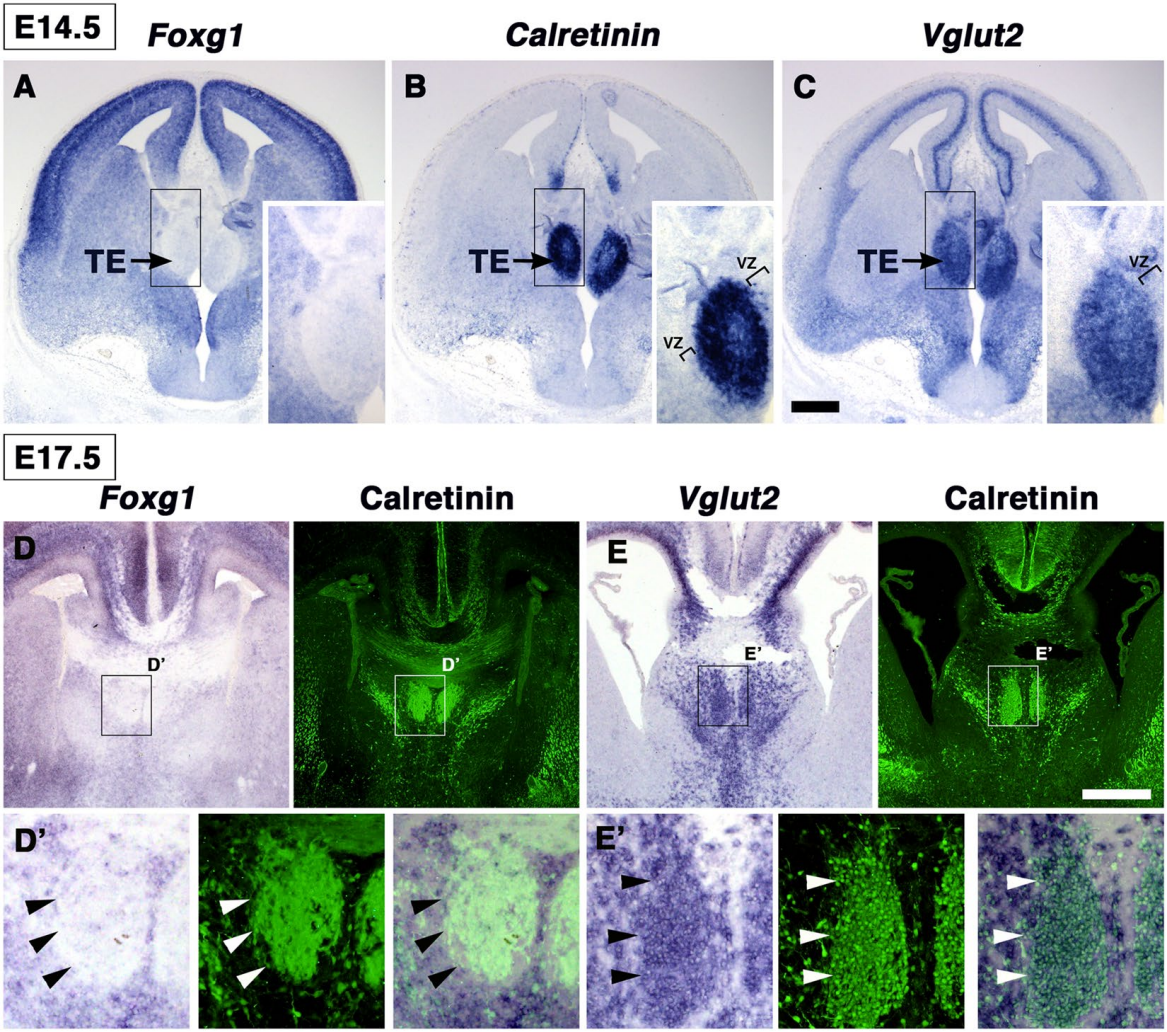
CalR-positive cell populations have a diencephalic identity. (**A**–**C**) ISH for *Foxg1* (**A**), *CalR* (**B**), and *Vglut2* (**C**) in the E14.5 forebrain. *Vglut2*, but not *Foxg1*, was expressed in the TE marked by *CalR* expression. (**D**,**E**) ISH for *Foxg1* (**D**) or *Vglut2* (**E**) followed by CalR immunostaining. CalR-expressing cell populations expressed *Vglut2*, but not *Foxg1* (arrowheads). Boxed areas in D and E are magnified in D’ and E’, respectively. TE, thalamic eminence; VZ, ventricular zone. Scale bars: 200 µm in A-C and 500 µm in D,E.

To study the extratelencephalic origin of *CalR*-positive cells in the developing septum, we examined the expression of *Foxg1*, a telencephalic marker gene^31^, which delineates the anterior border of the TE by repressing TE-related gene expression^32^. We confirmed that *Foxg1* expression was excluded from the E14.5 TE^32,33^ (Fig. 3A,B). Furthermore, *Foxg1* was negative in the CalR-expressing cells along the dorsoventral axis in the E17.5 telencephalon, as determined by ISH for *Foxg1* followed by CalR immunostaining (Fig. 3D’, arrowheads). A similar result was also observed in the P0 forebrain of *Foxg1*^*lacZ*/+^ mice (Supplementary Fig. S2). These results further suggested the extratelencephalic origin of the CalR-positive cells. We, therefore, examined the expression pattern of *Vglut2*, since both the TS and BAC are enriched in glutamatergic projection neurons^15,16^ (Fig. 1D,E). We found a dense distribution of *Vglut2*-positive cells in the mantle layer of the E14.5 TE (Fig. 3B,C). At E17.5, the distribution pattern of *Vglut2*, but not *Gad67*, overlapped with the CalR-positive cells in the developing septum (Fig. 3E’, Supplementary Fig. S3, arrowheads), indicating that these CalR-positive cells may be glutamatergic neuronal lineages. Taken together, these observations suggest that CalR-positive cells may take a rostrodorsal migratory path from the TE and may be destined to become TS and BAC neurons in the adult septum.

### TE-derived cells undergo rostrodorsal migration into the septum

**Figure 4:**
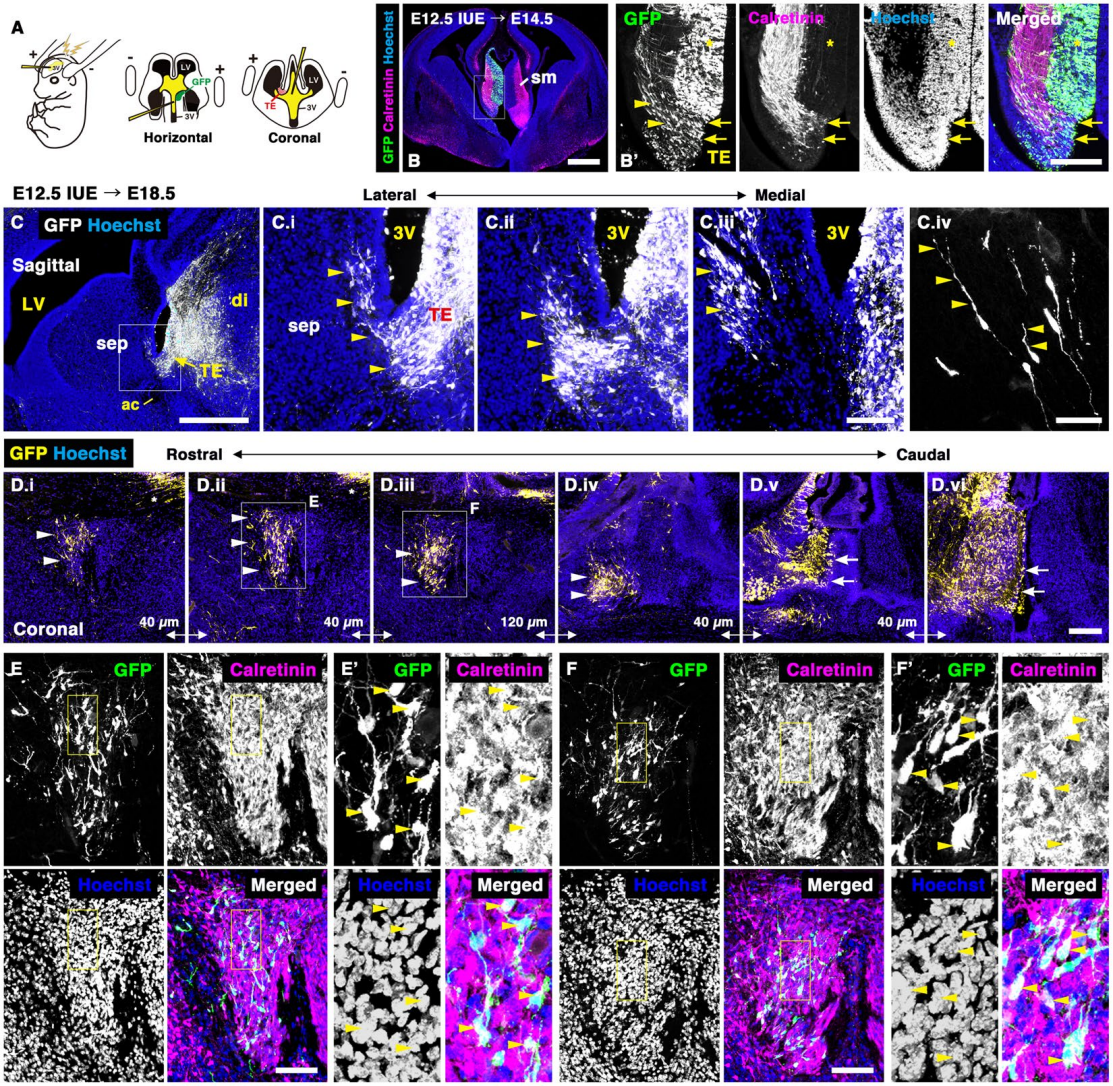
Thalamic eminence-derived cells undergo rostrodorsal migration into the septal nuclei. (**A**) Schematic diagram of in utero electroporation (IUE). Plasmid DNA was microinjected into the third ventricle of E12.5 brains and electroporated using a forceps-type electrode. (**B**) Coronal sections of E14.5 mouse embryos that were electroporated with pCX-GFP at E12.5. TE cells expressing CalR were labeled by GFP (arrows). (**C**,**D**) The TE was electroporated with pCX-EGFP at E12.5. The distribution of GFP-positive cells was analyzed in sagittal (**C**) and coronal (**D**) sections of E18.5 brains. The boxed area in C is magnified in C.i. C.iv shows migrating GFP-positive cells in the developing septum. D.i-D.vi show rostral (D.i) to caudal (D.vi) coronal sections. Many GFP-positive cells migrated rostrally from the TE to the septum. Arrows indicate the site of electroporation. (**E**,**F**) Confocal images of E18.5 brains stained with anti-GFP and anti-CalR antibodies. Boxed areas in E and F are magnified in E’ and F’, respectively. Almost all migrating GFP-positive cells expressed CalR, a marker of the TE (arrowheads). Sections were counterstained with Hoechst 33342. ac, anterior commissure; di, diencephalon; LV, lateral ventricle; sep, septal nuclei; sm, stria medullaris; TE, thalamic eminence; 3 V, third ventricle. Scale bars: 50 µm in C.iv, 100 µm in C.i-iii,E,F, 200 µm in B’,D, and 500 µm in B,C.

To demonstrate that the TE generates neurons of the posterior septal nuclei, we precisely traced the putative migratory path and fate of TE-derived cells by in utero electroporation (IUE). Both TS and BAC neurons are generated between E10 and E16, and their production peaks at E12-13^8,34^. Therefore, the TE of E12.5 mouse embryos was electroporated with pCX-EGFP plasmid, and two days to two weeks after electroporation, the distribution of GFP-positive cells was analyzed by immunostaining for GFP. To label the medial portion of the TE at the boundary between the telencephalon and diencephalon, DNA solution was injected into the third ventricle. After allowing a small amount of DNA to flow into the lateral ventricle, electropulses were applied (Fig. 4A). At E14.5, two days post-electroporation, cells in the ventricular zone (VZ) of the TE, identified with CalR immunostaining, were labeled by GFP (Fig. 4B’, arrows), and showed that many GFP-positive cells had migrated out from the VZ (Fig. 4B’, arrowheads). Under our experimental conditions, using 3-mm diameter disk electrodes, VZ cells of the diencephalon distributed dorsal to the TE were also labeled with GFP (Fig. 4B’, asterisk). Consequently, CalR, Tbr2, and Tbr1 were utilized as markers of TE-derived cells to distinguish TE cells from other cells produced in the dorsal part of the diencephalon as described below. At E18.5, six days after electroporation, many GFP-positive cells were found near the prospective septum, located anterior to the third ventricle (Fig. 4C.iii, arrowheads). The putative migratory stream from the TE to the septum was clearly observed in sagittal sections (Fig. 4C.i–C.iii, arrowheads). Most of the cells extended a single leading process rostrodorsally toward the emerging septum (Fig. 4C.iv, arrowheads), and some had an axon-like structure extending caudally. These observations indicated that GFP-positive cells migrated rostrally from the diencephalon including the TE, passed beneath the third ventricle, and migrated dorsally within the future posterior septal regions. Furthermore, coronal sections from the same stage revealed that a majority of the GFP-positive cells migrated rostrally, ipsilateral to the electroporation site (Fig. 4D.i–D.vi, arrowheads). To confirm that rostrally migrating GFP-positive cells are derived from the TE, we labeled them with CalR, which is expressed in the TE during embryogenesis and in the adult posterior septal nuclei^22,28^ (Figs 1 and 2). In the E18.5 prospective septal regions, after electroporation of GFP plasmid into the TE at E12.5, the majority of GFP-positive cells expressed CalR (Fig. 4E,F; CalR/GFP-double positive cells: 95.4 ± 6.5%, mean ± SD, n = 3). In many cases, GFP-positive cells were also observed in the hemisphere contralateral to the electroporation site resulting from DNA solution flowing into the lateral ventricle during electroporation (Fig. 4D.i,D.ii, Supplementary Fig. S4, asterisk). However, most of those cells, which were located in dorsolateral regions of the septum contralateral to the electroporated side, were negative for CalR when analyzed at E18.5 and P7 (Supplementary Figs S5 and S6, arrowheads).

**Figure 5:**
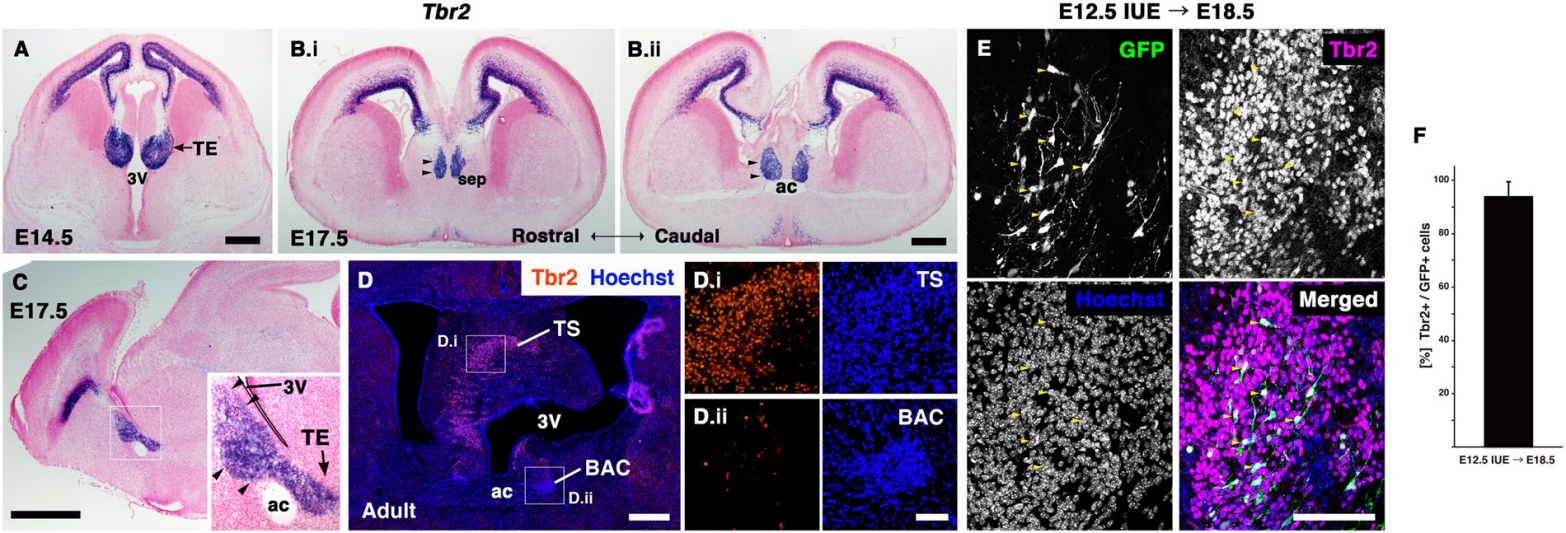
Tbr2 labeled the rostral stream from the TE. (**A**–**C**) *Tbr2* expression during development shown by ISH in E14.5 (**A**) and E17.5 (**B**) coronal sections, and at E17.5 in sagittal sections (**C**). *Tbr2* expression shows the continuous stream from the TE to the septal region, similar to CalR. (**D**) Immunostaining for Tbr2 in adult mouse brain. Boxed areas in **D** are magnified in D.i and D.ii. Tbr2 was expressed in the TS but not the BAC in the adult posterior septum. (**E**,**F**) Double immunostaining for GFP and Tbr2 at E18.5 in brains electroporated at E12.5 in the TE. E and F are images from different rostro-caudal levels. Most GFP-positive cells migrating rostrally colocalized with Tbr2 (arrowheads). Nuclei were labeled with Hoechst 33342. ac, anterior commissure; BAC, bed nuclei of the anterior commissure; sep, septal nuclei; TE, thalamic eminence; TS, triangular septal nucleus; 3 V, third ventricle. Scale bars: 100 µm in E, 500 µm in A,B,D, and 1 mm in C.

As mentioned above, diencephalic cells distributed outside of the TE were also labeled with GFP in our IUE experiments (Fig. 4B, asterisk). It is possible that these diencephalic cells in more dorsal areas, excluding the TE, migrated to the septal regions. Therefore, we next focused on the expression of the transcription factors Tbr2 and Tbr1. As described previously, *Tbr2* is strongly expressed in the TE at E14.5^35,36^ (Fig. 5A). Furthermore, in the E17.5 developing septal regions, *Tbr2* expression showed a dorsoventral distribution pattern, similar to that of *CalR* (Fig. 5B,C, arrowheads). Interestingly, *Tbr2* expression was highly restricted to the population of CalR-positive cells around the septum. In addition, we found that TS neurons, but not BAC neurons, expressed Tbr2 in adult mouse brains (Fig. 5D, Supplementary Fig. S7). These observations indicate that Tbr2 is a useful marker for tracing TE-derived cell lineages during septal formation. The E12.5 TE was labeled with GFP and migrating GFP-positive cells were analyzed at E18.5 by double-staining for GFP and Tbr2. Almost all GFP-positive cells expressed Tbr2 in the prospective septum as observed for CalR (Fig. 5E,F; Tbr2/GFP-double positive cells: 94.1% ± 6.5%, mean ± SD, n = 3). We observed similar patterns for Tbr1 staining^36,37^ (Supplementary Fig. S8). These results suggest that rostrally migrating GFP-positive cells originated from the TE.

**Figure 6:**
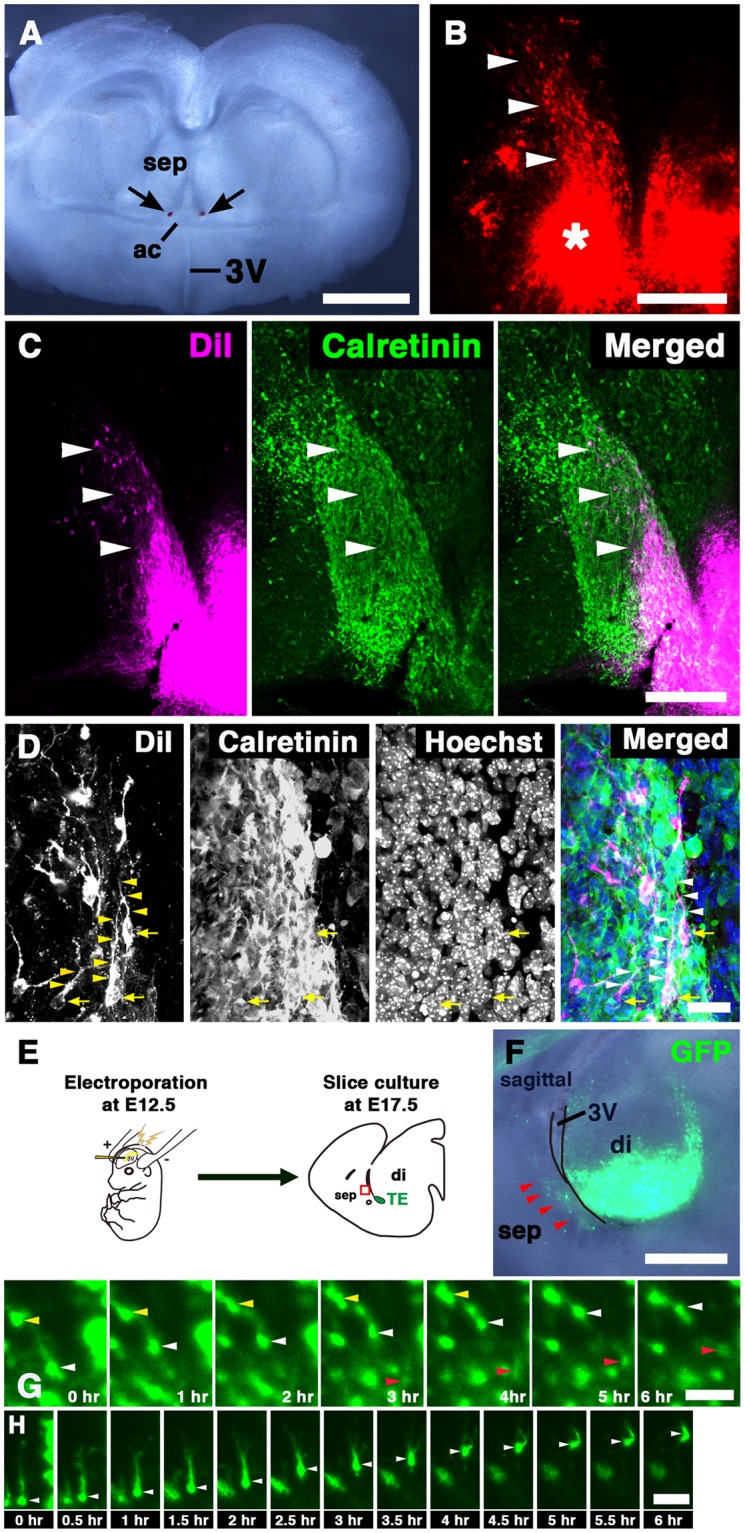
Migration of TE-derived cells in forebrain slices. (**A**) Bright-field images of E16.5 forebrain slices. DiI crystals were inserted within the putative migratory pathway of CalR-positive cells (arrows). (**B**) After incubation for 24 hrs, many DiI-labeled cells (arrowheads) were observed dorsal to the DiI application site (asterisk). (**C**,**D**) Immunostaining for CalR in vibratome sections of DiI-labeled slices. DiI-labeled cells were observed in the putative migratory pathway of CalR-positive cells (arrowheads in **C**). Many of them had a dorsally extended leading process (arrowheads in **D**). Yellow arrows indicate cell bodies. (**E**) Schematic diagram of slice culture. (**F**) Sagittal forebrain slices electroporated with GFP plasmid in the TE. (**G**,**H**) Time-lapse imaging of GFP-positive cells in the septal region. Arrowheads indicate dorsally migrating GFP-positive cells. ac, anterior commissure; di, diencephalon; sep, septal nuclei; 3 V, third ventricle. Scale bars: 25 µm in D, and 50 µm in G,H, 200 µm in B,C, and 1 mm in A,F.

To further examine this migration, we performed *in vitro* DiI labeling of the putative migrating cells. E16.5 forebrain slices, in which small DiI crystals were inserted at a location dorsal to the anterior commissure (Fig. 6A), were cultured for 24 hrs, and subsequently stained with an anti-CalR antibody. Many DiI-labeled cells, which had migrated dorsally from the DiI application site, were observed in the developing septum (Fig. 6B, arrowheads; n = 4). In addition, vibratome sections of the forebrain slices were also stained with the anti-CalR antibody. Most of the DiI-labeled cells in these sections were located within the population of CalR-positive cells (Fig. 6C, arrowheads), which is the putative migratory pathway of TE-derived cells (Fig. 2E.ii). When observed at high magnification, many DiI-labeled cells had a single leading process extending dorsally, and some also showed the typical bipolar morphology of migrating neurons (Fig. 6D, arrowheads). To directly observe their migration, we performed time-lapse imaging. The E12.5 TE was electroporated with GFP, and sagittal forebrain slices prepared at E17.5 were cultured (Fig. 6E). We then observed the behavior of GFP-positive cells distributed throughout the septal area (Fig. 6F, arrowheads). Some of these GFP-positive cells migrated dorsally toward the future posterior septum during the 6 hour imaging period (Fig. 6G,H, arrowheads; Movies 1, 2; n = 3). Taken together, these results suggest that TE-derived cells leave the diencephalon and enter the prospective septal regions through rostrodorsal migration.

### The thalamic eminence is the origin of TS and BAC neurons

**Figure 7:**
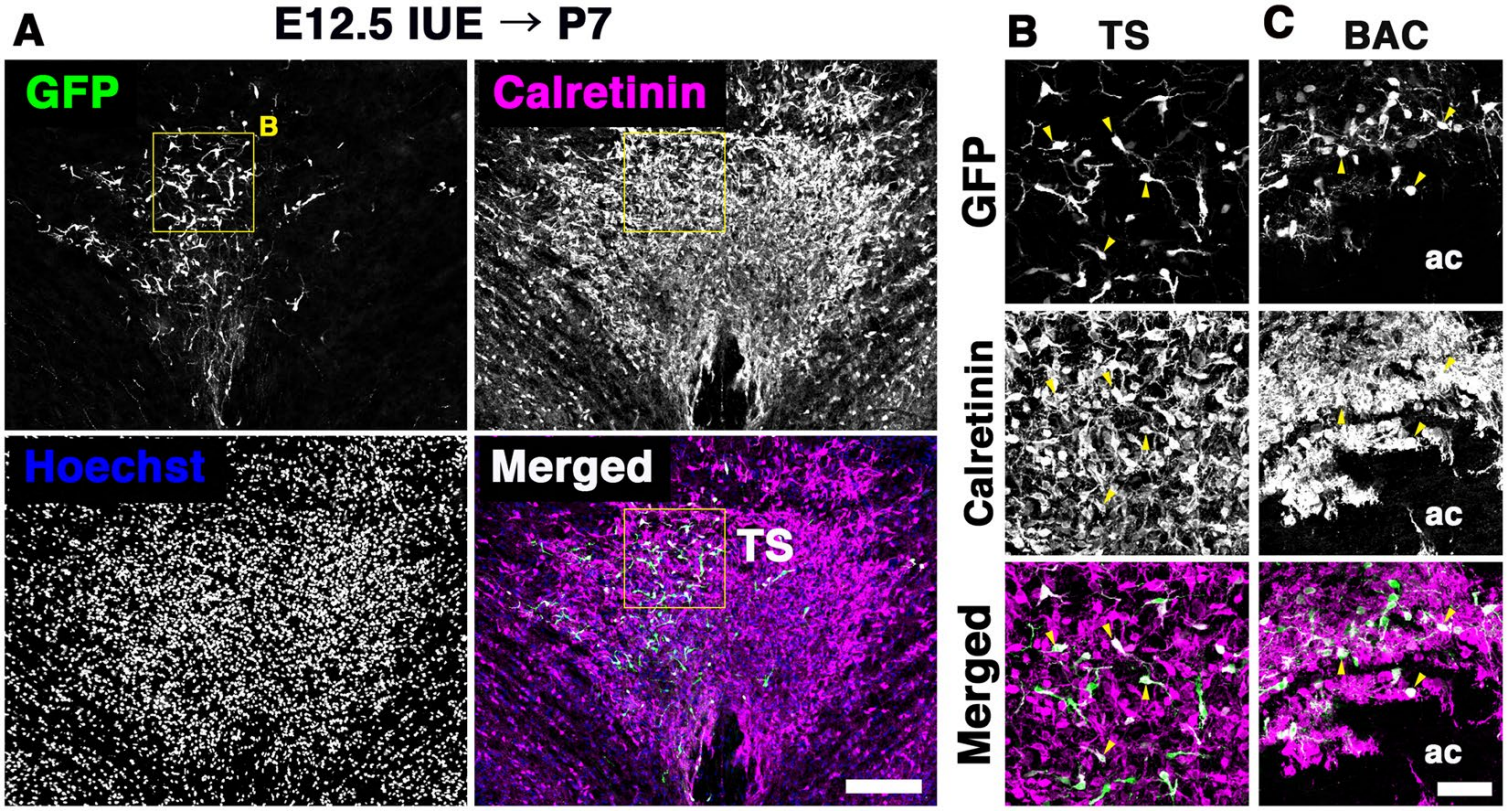
The thalamic eminence is a source of both TS and BAC neurons. The TE was labeled with GFP by in utero electroporation at E12.5. Distribution of GFP-positive cells was analyzed in the TS and BAC at P7. Figures show double immunostaining for GFP and CalR. Sections were counterstained with Hoechst 33342. Both TS and BAC neurons were labeled with GFP in the electroporated postnatal brains (arrowheads). ac, anterior commissure; BAC, bed nuclei of the anterior commissure; TS, triangular septal nucleus. Scale bars: 200 µm in A and 50 µm in B,C.

We next examined whether TE-derived cells, which migrate rostrally, are destined to reside in both the TS and BAC. The distribution of GFP-positive cells was analyzed at P7, two weeks after electroporation into the TE. A number of GFP-positive cells were distributed in both the TS and BAC at P7, many of which were observed ipsilateral to the electroporation site (Fig. 7A). Most of these expressed CalR, although the expression level of CalR varied (Fig. 7B,C; CalR/GFP-double positive cells: TS, 94.1% [239/254, n = 4], BAC, 90.3% [130/144, n = 3]). These results suggest that the posterior septal neurons in both the TS and BAC arise from the TE through rostrodorsal migration.

### Putative migratory pathway of TE-derived septal neurons along the fornix

**Figure 8:**
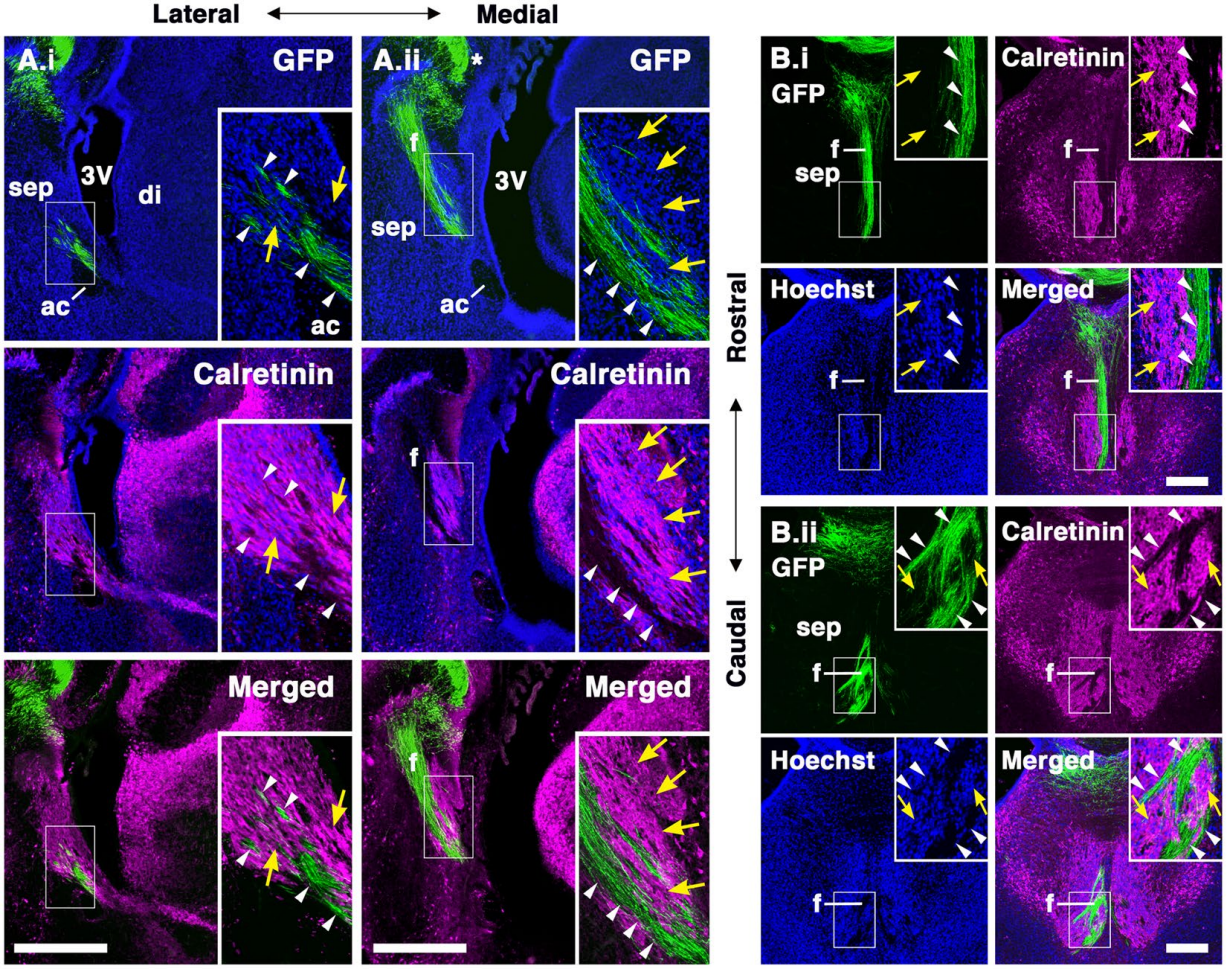
Putative migratory pathway of TS and BAC neurons along the fornix. (**A**,**B**) The fornix was labeled by in utero electroporation into the E12.5 medial cortex. Embryos were allowed to develop until E18.5 and brain sections were immunostained for GFP and CalR; (**A**) sagittal sections, (**B**) coronal sections. CalR-expressing cells (yellow arrows) was distributed along the fornix (arrowheads) labeled by GFP at the caudal region of their putative migratory trajectory. The asterisk indicates the site of electroporation. Nuclei were labeled with Hoechst 33342. ac, anterior commissure; di, diencephalon; f, fornix; sep, septal nuclei; sm, stria medullaris; 3 V, third ventricle. Scale bars: 200 µm in B and 500 µm in A.

The stream of CalR-positive cells was more clearly observed at the medial level at E17.5, whereas the TE is located more laterally (Supplementary Fig. S9), indicating that TE-derived cells appeared to migrate rostrally in a lateral-to-medial trajectory from the TE to the septum. Furthermore, these CalR-positive cells overlapped with the axonal tracts labeled by neurofilament (Supplementary Fig. S9). The fornix is a major output tract from the hippocampus to the septum and mammillary nuclei of the diencephalon. Their axons pass through the medial portion of the developing septum^38^. These observations raised the possibility that the migratory path of TE-derived CalR-positive cells is adjacent to the fornix. To examine this, the fornix was labeled by IUE into the E12.5 dorsomedial cerebral cortex including the hippocampal primordium. At E18.5, the fornix was clearly labeled by GFP immunostaining (Fig. 8A,B). The GFP-positive axons extended ventrally, close to the midline of the septum from the electroporated site (Fig. 8A,B). In sagittal sections, the axons directed toward the caudal regions of the diencephalon could be observed (Fig. 8A, arrowheads). In the presumptive TS region, the CalR-expressing cell population was distributed caudal to the fornix (Fig. 8A.ii, yellow arrows). At the rostral level in coronal sections, CalR-positive cells appeared to be adjacent to but segregated from the fornix (Fig. 8B.i). In contrast, at more caudal levels, the CalR cell clusters and the fornix were intermingled (Fig. 8A.i,B.ii). These observations suggest that TE-derived posterior septal neurons migrate rostrally along the fornix and subsequently detach from the axonal tract around the prospective septal regions.

## Discussion

**Figure 9:**
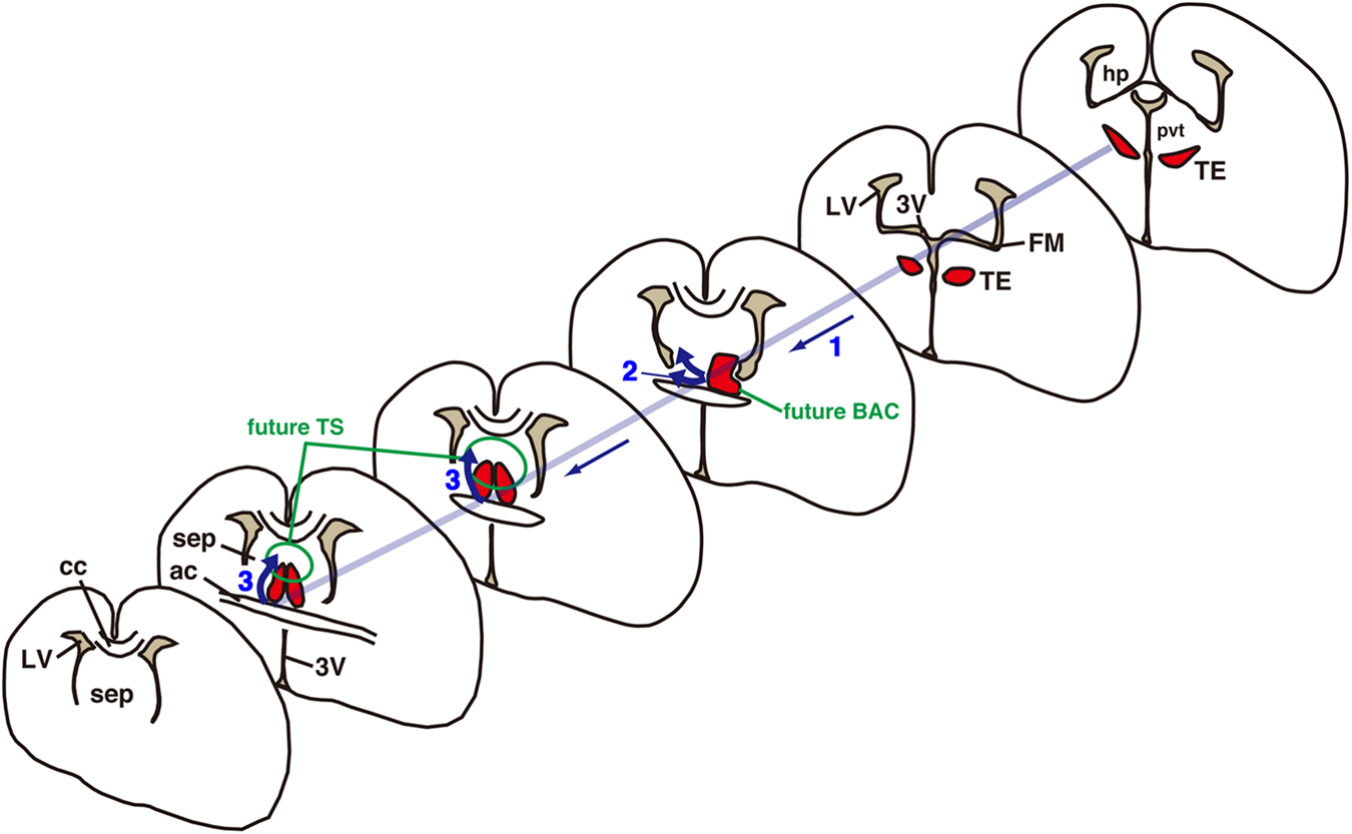
Schema of the putative migratory pathway of TE-derived posterior septal neurons. Both TS and BAC neurons originate from the TE of the diencephalon and migrate rostrally toward the telencephalic region (1). BAC neurons appear to terminate their migration at locations caudal to the anterior commissure (2). In contrast, TS neurons continue their migration dorsally toward prospective TS regions (3). ac, anterior commissure; BAC, bed nuclei of the anterior commissure; cc, corpus callosum; hp, hippocampus; LV, lateral ventricle; pvt, paraventricular thalamic nucleus; sep, septal nuclei; TE, thalamic eminence; TS, triangular septal nucleus; 3 V, third ventricle.

In this study, we addressed the origin of the two posterior septal nuclei, the TS and BAC. We identified a new rostrodorsal migratory stream from the thalamic eminence (TE) to the posterior septal nuclei by combining marker expression patterns and in utero electroporation. Tracing of progenitors in the TE revealed that both TS and BAC neurons originated from the TE. Furthermore, we found that their migration path occurred along the fornix. Taken together, our results indicate that neurons in the posterior septal nuclei have a distinct origin from other septal nuclei and, during development, travel a long distance from the diencephalic to telencephalic territory along the hippocampal axons (Fig. 9).

The septum is subdivided into several regions^1^. Neurons in the LS and MS originate from the neuroepithelium of the ventromedial wall of the lateral ventricle and from other subpallial regions, such as the MGE and POA, indicating their multiple telencephalic origins^8,9^. In this study, we identified a novel progenitor pool of posterior septal neurons in the TE, which is considered to be a diencephalic embryonic structure^18–20,22^. Newly born posterior septal neurons migrated rostrally into the telencephalic territory beyond the diencephalic-telencephalic boundary (Figs 2, 4 and 6). Thus, neural progenitors in both the telencephalon and diencephalon contribute to the formation of the mammalian septal nuclear complex.

It is unknown if all TS and BAC neurons originate from the TE. However, we observed a large number of CalR-positive cells forming a rostrodorsal stream (Fig. 2E,F). Furthermore, the stream could be observed from late embryonic to early postnatal stages (Fig. 2). These observations suggest that many TE cells contribute to the formation of both the TS and BAC. However, we could not exclude the possibility that other telencephalic and diencephalic progenitors contribute to the posterior septal nuclei. In contrast to the TS and BAC, few GFP-positive TE cells were observed in either the postnatal MS or LS, although many cells were distributed in the SFi (Fig. 7). This suggests that the TE does not produce MS and LS neurons, and consistent with this, it has been reported that both the MS and LS have their origins in the telencephalic territory^8,9^.

The adult TS and BAC were shown to be enriched with glutamatergic neurons^15^ (Fig. 1D,E). Other septal nuclei, such as the MS, also contain glutamatergic neurons^39^. Many TE-derived cells expressed the glutamatergic neuron marker, *Vglut2*, although few TE-derived cells were observed in the LS and MS as described above (Fig. 3C,E, Supplementary Fig. S6). Furthermore, we observed other populations of *Vglut2*-positive cells from the VZ of the medial wall of the lateral ventricle to the septal region (Fig. 3E). These observations suggest that glutamatergic neurons in the LS and MS are primarily generated from local progenitors in the telencephalon.

We observed many GFP-positive cells in both the postnatal TS and BAC in which the TE was electroporated at E12.5 (Fig. 7), indicating that the TE is an origin of both TS and BAC neurons. During development, TE-derived cells destined to become TS and BAC neurons are born at the same developmental stages^34^ and migrate rostrally toward the telencephalon (Figs 2, 4 and 6). Subsequently, only BAC neurons may terminate their migration at locations caudal to the anterior commissure. In contrast, TS neurons appear to continue their migration dorsally along the fornix and detach from the axonal bundle near their final destination (Figs 4, 8 and 9). At present, we do not know whether the fornix acts as a scaffold for migrating TE-derived cells, nor what factors act as a stop signal for migrating TS and BAC neurons. However, it is possible that secreted and/or cell adhesion proteins may be involved in interactions between migrating TE cells and fornical axons, as observed in the radial migration of cortical neurons^40,41^.

The septal nuclei have reciprocal connections with the hippocampus through the fornix, and the TS and BAC also receive afferents from the hippocampus^1,2,5^. The migration of TE cells along the fornix is suggestive of their direct interaction from developmental stages. In contrast, TS and BAC neurons send their axons dorsocaudally to the MHb^3^. We found that migrating TE cells had a caudal axon-like structure (Fig. 4C.iv), suggesting that their cell bodies migrate rostrally to the septum, while extending their axons behind. The molecular mechanisms underlying the migration and axonal extension of posterior septal neurons have been unexplored. Ephrin/Eph signaling has been suggested to be involved in topographic projections in the septo-hippocampal circuit^42^, and *Ephas* are expressed in the developing TE^43^. Thus, Ephrin/Eph signaling may regulate neural migration of posterior septal neurons along the fornix during development.

The TE is a transient developmental structure during mammalian embryogenesis, whereas it is prominent through adulthood in lower vertebrates^22,44–46^. However, the fate and function of the mammalian TE is not fully understood. Several studies have proposed that the TE acts as a signaling center in the developing forebrain^21,22^. The TE, which expresses high levels of Wnt family members, has the capacity to induce fate changes of progenitors in the ventral telencephalon *in vitro*^47^. In contrast, TE-derived cells appear to disperse throughout the forebrain and differentiate into various cell types, such as Cajal-Retzius cells, pAOB neurons, lot cells, as well as posterior septal neurons^25,26^ (Figs 4 and 7). However, posterior septal neurons appear to be born later than Cajal-Retzius cells and pAOB neurons^25,34^. While TS and BAC neurons are generated from the medial part of the TE, which is a neuroepithelium flanked by the third ventricle (Fig. 4), lot cells are born in the lateral part of the TE, located in close proximity to the choroid plexus^26^. Therefore, it is reasonable to hypothesize that specific neurons derived from the TE may have distinct spatiotemporal origins within the TE. Although it is unclear why such varied neurons in different forebrain regions originate in the TE, the mammalian TE presumably generates neurons related to the olfactory processing system and emotion-related behaviors, such as fear and anxiety^4,25,26^ (Fig. 4). In non-mammalian vertebrates, the TE is interconnected with several secondary olfactory centers, suggesting its involvement in the processing of olfactory information^45^. It is possible that TE-derived cells have evolutionarily conserved functions^25^. In conclusion, neural progenitors of the diencephalic TE may contribute to neuronal diversity in various brain regions, including the septal nuclei, of the mammalian telencephalon.

## Materials and Methods

### Animals

Timed-pregnant and adult mice (Slc:ICR) were obtained from Japan SLC. *Foxg1*^*lacZ*/+^ mice were maintained on a CD1 background^31,32^. They were housed on a normal 12 h light/dark schedule with free access to food and water. Noon on the day of the vaginal plug was considered embryonic day 0.5 (E0.5). The day of birth was defined as postnatal day 0 (P0). All procedures were performed in accordance with protocols approved by the Animal Research Committee of Niigata University and the Animal Experimentation Committee of Waseda University. All efforts were made to minimize animal suffering.

### Immunohistochemistry

P0 or older mice were perfused transcardially with 4% paraformaldehyde (PFA) in PBS after euthanasia with an intraperitoneal injection of sodium pentobarbital (125 mg/kg body weight). Pregnant females were euthanized with sodium pentobarbital, and embryos were isolated in cold 0.01 M PBS (pH 7.4). Dissected postnatal or fetal brains were fixed by immersion in 4% PFA overnight at 4 °C. Then, brain samples were embedded in paraffin wax (Leica Biosystems) or in OCT compound (Sakura) according to the application. Frozen sections were cut at 20 µm thickness on a cryostat (HM560; Micron), then mounted onto MAS-coated glass slides (Matsunami). Paraffin sections were cut at 9 µm thickness using a rotary microtome. Paraffin sections were used for *Calretinin in situ* hybridization, and frozen sections were used for other staining. Immunohistochemistry^48^ and X-gal staining^49^ were performed as previously described. The following primary antibodies were used: rabbit anti-Calretinin (CalR, 1:1000, Swant), rat anti-GFP (1:2000, Nacalai Tesque), mouse anti-Neurofilament M (2H3, 1:50, DSHB), rabbit anti-Tbr1 (1:500, Abcam), and rabbit anti-Tbr2 (1:500, Abcam). For immunofluorescence, sections were labeled with species-specific secondary antibodies conjugated to Alexa Fluor 488 or 594 (Invitrogen) and counterstained with Hoechst 33342 (Sigma). Confocal images were captured with a confocal laser scanning microscope (FV1200, Olympus). Pictures were further processed with Photoshop CS4 (Adobe) for general adjustment of contrast and brightness or for conversion to grayscale images.

### *In situ* hybridization (ISH)

*In situ* hybridization (ISH) was performed as described previously^50^. The following cDNAs were generated by RT-PCR, then subcloned into the pCRII-TOPO vector (Invitrogen) to generate RNA probes. The primer sequences used for ISH are as follows: *Calretinin* (934 bp), 5′-CCAGTTCCTGGAAATCTGGA-3′ and 5′-AGGCCTAAGGAACCCTACCA-3′; *Foxg1* (984 bp), 5′-TGATTCCCAAGTCCTCGTTC-3′ and 5′-TTTGAGTCAACACGGAGCTG-3′; *Gad67* (*Gad1*; 980 bp), 5′-TGAACCGTAGAGACCCCAAG-3′ and 5′-CCCCCTTTCATTGCACTTTA-3′; *Tbr1* (939 bp), 5′-GTTCTAGCGCACTCGCTCTT-3′ and 5′-GCACTTTGGGGAAAAACAAA-3′; *Tbr2* (814 bp), 5′-GGCAAAGCGGACAATAACAT-3′ and 5′-TTTCCTTGGCAAGCTGATCT-3′; *Vglut2* (*Slc17a6*; 1110 bp), 5′-GGTTCGATGACGTTTCTGGT-3′ and 5′-GCAATGACTGCTCCAGCATA-3′. DIG-labeled RNA probes were reacted with alkaline phosphatase-conjugated anti-DIG antibody (Roche). Reaction product was visualized by incubating the sections with nitroblue tetrazolium chloride (NBT; Roche) and 5-bromo-4-chloro-3-indolylphosphate (BCIP; Roche). Sections were counterstained with Nuclear Fast Red. Pictures were taken with a digital camera (DP72, Olympus). Results shown are representative of at least three independent experiments.

### In utero electroporation (IUE)

Timed-pregnant mice were deeply anesthetized with isoflurane. Plasmid DNA (1–2 µg/µl, pCX-EGFP) was microinjected into the third or lateral ventricles of E12.5 brains and electroporated using an electroporator (Fig. 4A; five 50 millisecond pulses of 50 V for the TE or 40 V for the medial cortex, with an interval of 950 milliseconds; CUY21, NEPA GENE) with a forceps-type electrode (CUY650P3). Embryos or postnatal mice were dissected 48 hours to 2 weeks after electroporation. All data were obtained from at least three independent experiments.

### Slice culture

E16.5 mouse brains were dissected and embedded in 3% low-melting point agarose. Coronal forebrain slices (350 µm) were cut using a vibratome (Dosaka EM). The slices were placed onto Millicell culture inserts (Millipore) that were put into 35 mm diameter dishes and incubated with 1.2 ml Neurobasal medium supplemented with B27, 2 mM Glutamax and Penicillin-Streptomycin (Invitrogen) at 37 °C under 5% CO_2_. To label the migratory pathways of TE-derived cells, small DiI crystals (1,1′-dioctadecyl-3,3,3′,3″-tetramethylindocarbocyanine perchlorate; Invitrogen) were inserted in the region dorsal to the anterior commissure. After incubation for 24 hours, the slices were fixed with 4% PFA and cut with a vibratome at 40 µm thickness. The sections were immunostained with anti-CalR antibody without detergents and observed with a confocal laser scanning microscope (FV1200). For time-lapse imaging, sagittal forebrain slices (350 µm) were prepared from E17.5 brains in which the TE had been electroporated at E12.5 as described above. Four optical Z-sections imaged at 15 µm intervals were captured every 15 minutes for 6 hours using the FV1200.

### Quantification

To quantify the percentage of CalR- or Tbr2-positive cells among the GFP-labeled cells, confocal images were captured using a 20x objective from both the TS and BAC of E18.5 or P7 brains, which had been electroporated with the GFP plasmid at E12.5 in the TE. A total of 188 and 276 GFP-labeled cells at E18.5 were counted for quantification of CalR/GFP and Tbr2/GFP double positive cells, respectively, in at least three sections from three different animals.

## Electronic supplementary material

supplementary information

Movie 1

Movie2
