# Supplementary material for: Diencephalic progenitors contribute to the posterior septum through rostral migration along the hippocampal axonal pathway
Source: Sci Rep. 2018 Aug 6;8:11728. doi: 10.1038/s41598-018-30020-9 (PMC6078977; doi:10.1038/s41598-018-30020-9)

**Supplemental information**

**Diencephalic progenitors contribute to the posterior septum through rostral migration along the hippocampal axonal pathway**

Keisuke Watanabe, Koichiro Irie, Carina Hanashima, Hirohide Takebayashi and Noboru Sato

**Legends for supplemental figures**

**Figure S1.** Expression pattern of *Calretinin* in the E14.5 mouse brain.

Coronal sections stained for *Calretinin* (*CalR*) shown from rostral (A.i) to caudal (A.v) in the E14.5 forebrain. Figure 2C shows a more caudal section than Figure S2A.v. At E14.5, the thalamic eminence (TE) was identified by *CalR* expression. (B) Sagittal image from an E14.5 brain. sep, septal nuclei; TE, thalamic eminence; 3V, third ventricle. Scale bars: 500 µm.

**Figure S2.** X-gal staining in the P0 *Foxg1^lac Z/+^* mice.

DAPI (A) and X-gal staining (B) in the P0 forebrain of *Foxg1^lacZ/+^* mice. The cell population distributed along the dorsoventral axis was not labeled by X-gal staining (arrowheads). The boxed area in A and B is magnified in A’ and B’, respectively. Scale bars: 200 µm in B and 500 µm in A.

**Figure S3.** The populations of CalR-positive cells did not express the GABAergic neuron marker.

(A) ISH for *Gad67* followed by CalR immunostaining. Few CalR-positive cells expressed *Gad67*, a marker for GABAergic neurons (arrowheads). The boxed area is magnified in A’. Scale bars: 500 µm.

**Figure S4.** The contralateral cortical hem labeled by in utero electroporation.

E12.5 mouse brains were electroporated with GFP plasmid and analyzed at E14.5. Diencephalic regions including the TE were labeled by GFP (arrows). The contralateral cortical hem was also labeled by GFP due to the flow of DNA solution from the third ventricle to the lateral ventricle (asterisk).

**Figure S5.** GFP-positive cells on the side contralateral to the electroporation.

The E12.5 TE was electroporated with the pCX-EGFP plasmid, and GFP-positive cells in the E18.5 septal region were observed by immunostaining for GFP and CalR. Images were obtained in the region close to the lateral ventricle on the side contralateral to the electroporation. Few GFP/CalR-double positive cells were observed in the contralateral dorsal septal regions (arrowheads). Nuclei were labeled with Hoechst 33342. Scale bars: 100 µm.

**Figure S6.** GFP-positive cells derived from the side contralateral to the electroporation were negative for Calretinin.

The GFP plasmid was electroporated into the TE at E12.5 and embryos were allowed to develop until P7. Figures show double-staining for GFP and CalR. Boxed areas in A are magnified in B. Most GFP-positive cells located on the side contralateral to the in utero electroporation were negative for CalR (arrowheads). Nuclei were labeled with Hoechst 33342. TE, thalamic eminence. Scale bars: 100 µm in B and 500 µm in A.

**Figure S7.** *Tbr2* expression in the adult septal nuclei.

Expression of *Tbr2* visualized by ISH in the adult brain. TS neurons highly expressed *Tbr2* mRNA. LS, lateral septal nucleus; TS, triangular septal nucleus. Scale bar: 500 µm.

**Figure S8.** Tbr1 labeled the cohorts of TE-derived cells.

(A-C) *Tbr1* expression during development shown by ISH in coronal sections at E14.5 (A) and E17.5 (B). In the TE and developing septal region, the expression pattern of Tbr1 was similar to that of Tbr2 (arrowheads). (D,E) Double immunostaining for GFP and Tbr1 in the E18.5 brain electroporated at E12.5 in the TE. GFP-positive cells were colocalized with Tbr1 (arrowheads). Nuclei were labeled with Hoechst 33342. sep, septal nuclei; TE, thalamic eminence. Scale bars: 100 µm in C and 500 µm in A,B.

**Figure S9.** CalR-positive cells along the axonal tract.

(A) Immunohistochemistry for CalR in lateral (A.i) to medial (A.iii) sagittal sections from the E17.5 forebrain. Nuclei were labeled by Hoechst 33342. (B) Double immunostaining for CalR and neurofilament (NF-M). CalR-positive cells (yellow arrows) were distributed along axonal tracts (arrowheads) labeled by neurofilament. Boxed areas in B are magnified in B’. ac, anterior commissure; di, diencephalon; f, fornix; sep, septal nuclei; sm, stria medullaris; 3V, third ventricle. Scale bars: 50 µm in B’, 200 µm in B, and 500 µm in A.

**Movie 1.** Dorsal migration of GFP-positive cells in the septal area.

A sagittal forebrain slice was prepared from a brain electroporated with GFP plasmid in the TE. Migratory behavior of GFP-positive cells was observed in the septal area.

**Movie 2.** Dorsal migration of GFP-positive cells in the septal area.

Another representative sample of a sagittal forebrain slice prepared from a brain electroporated with GFP plasmid in the TE. Migratory behavior of GFP-positive cells was observed in the septal area.


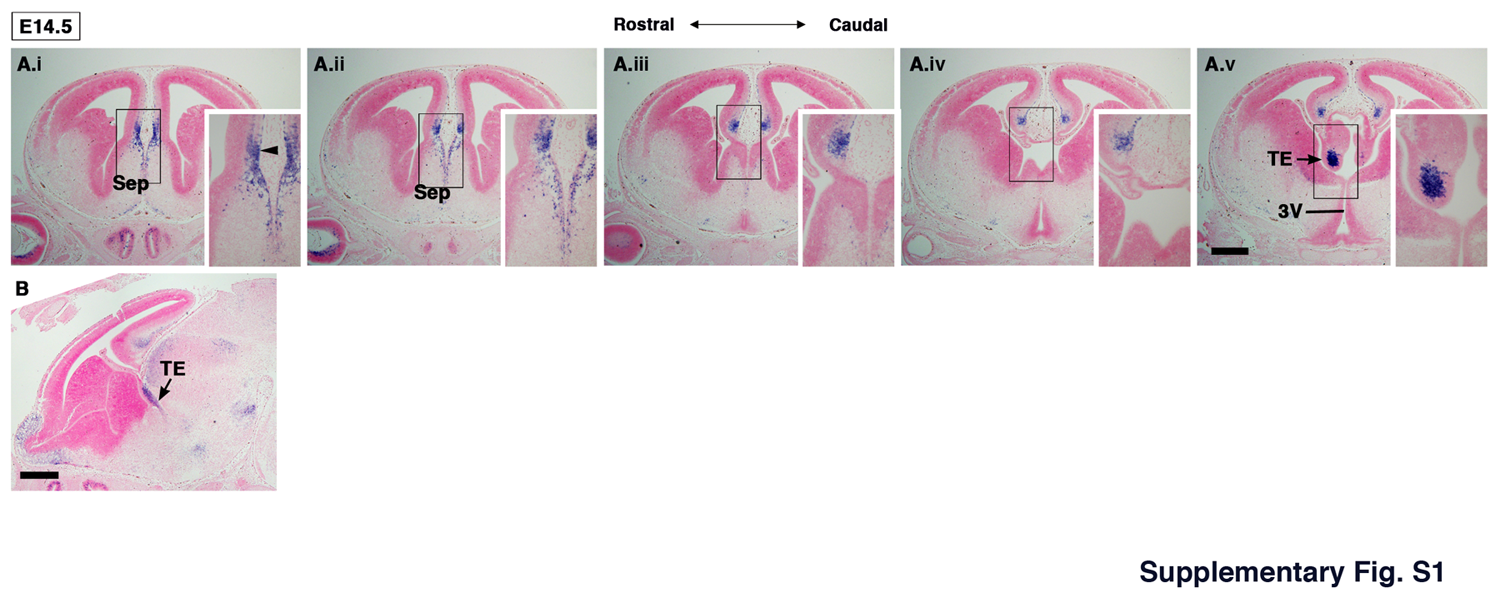


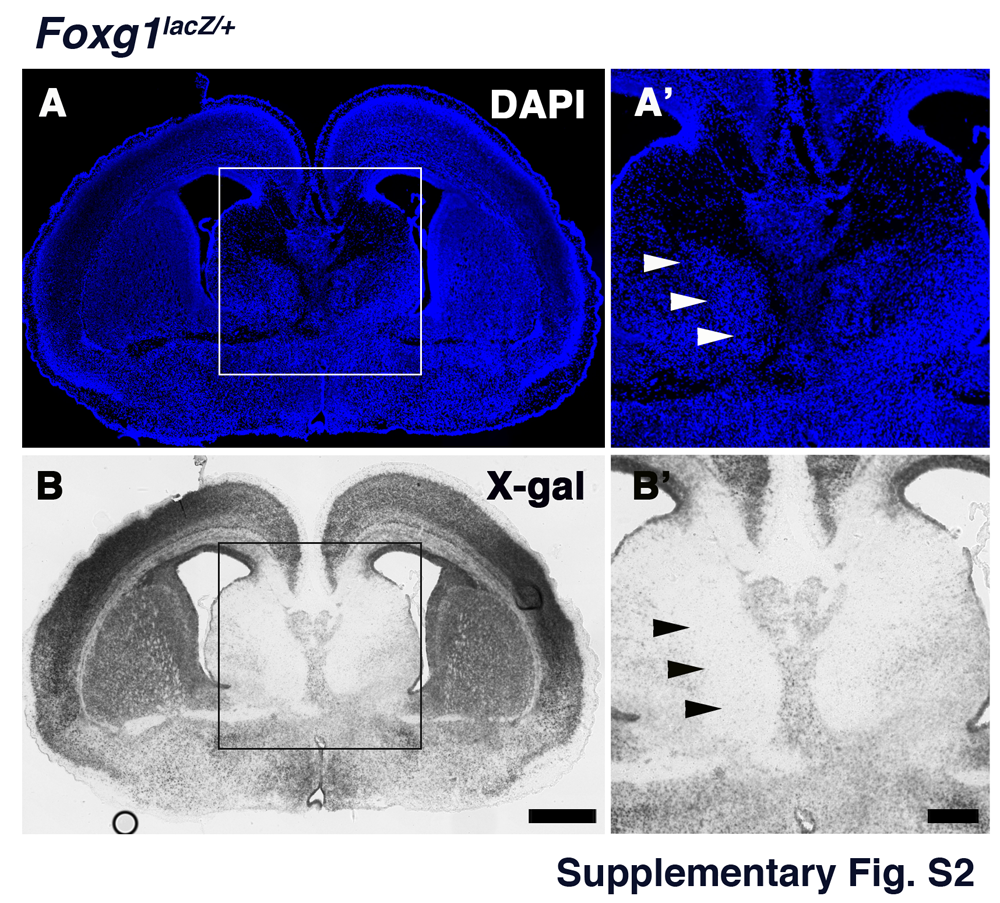


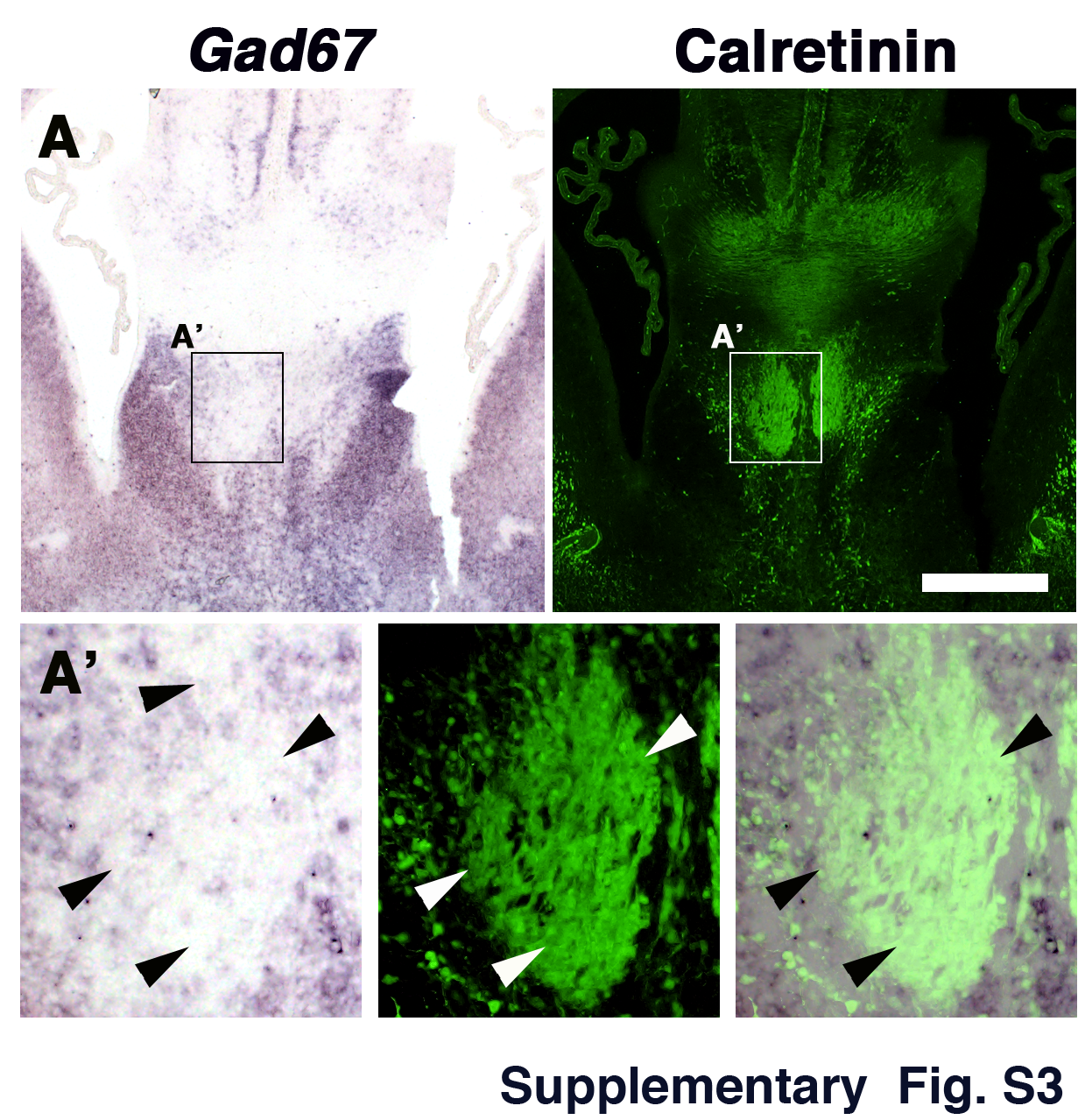


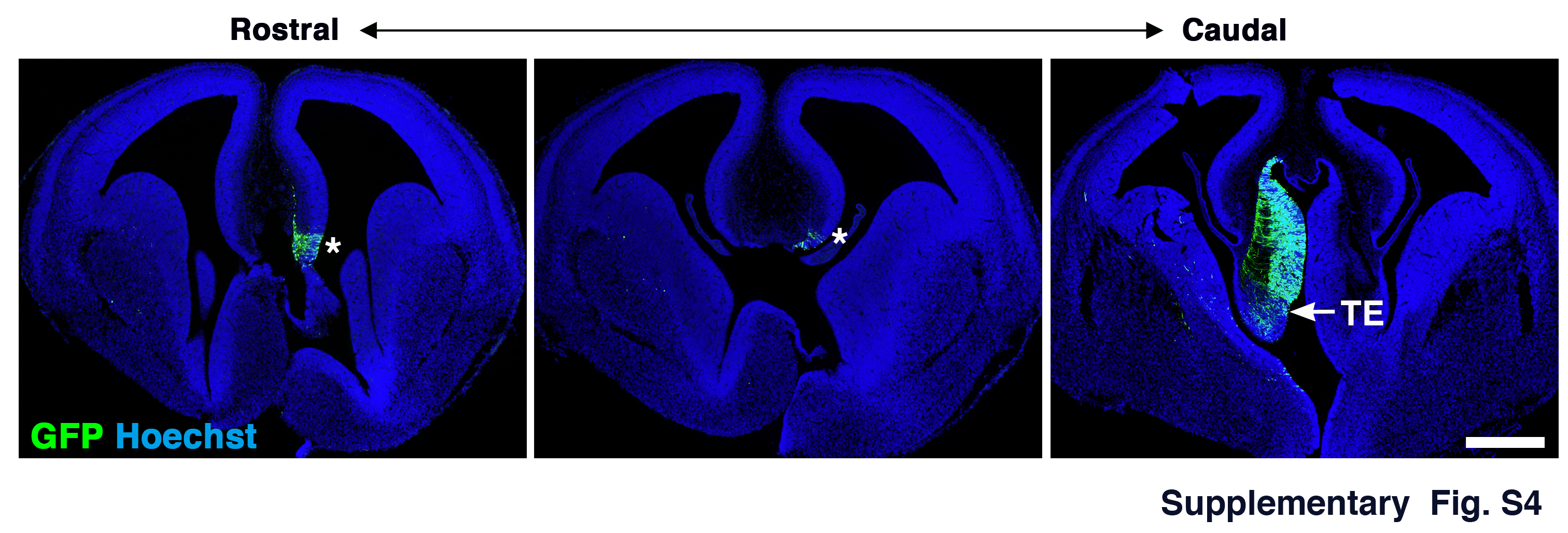


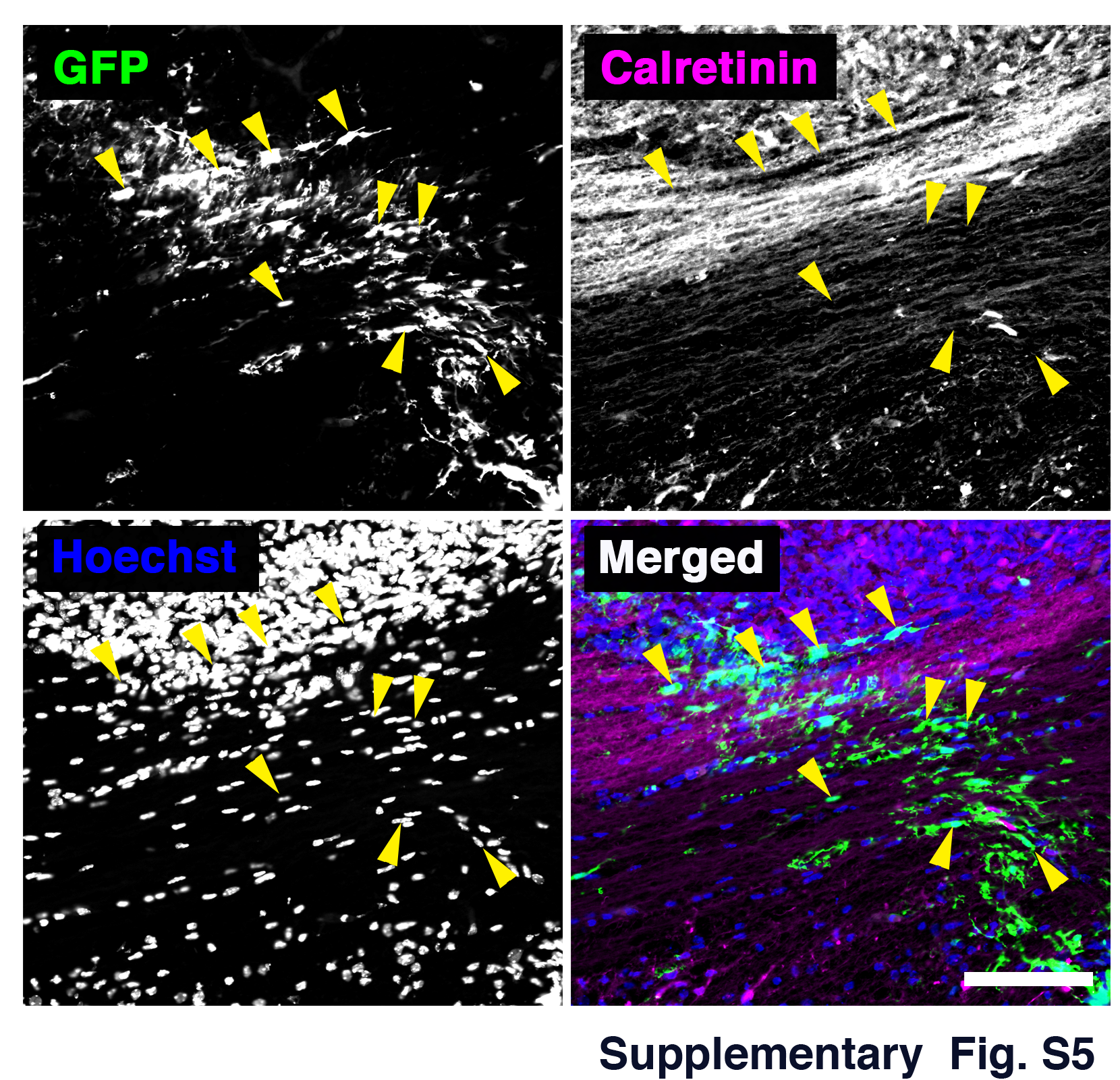


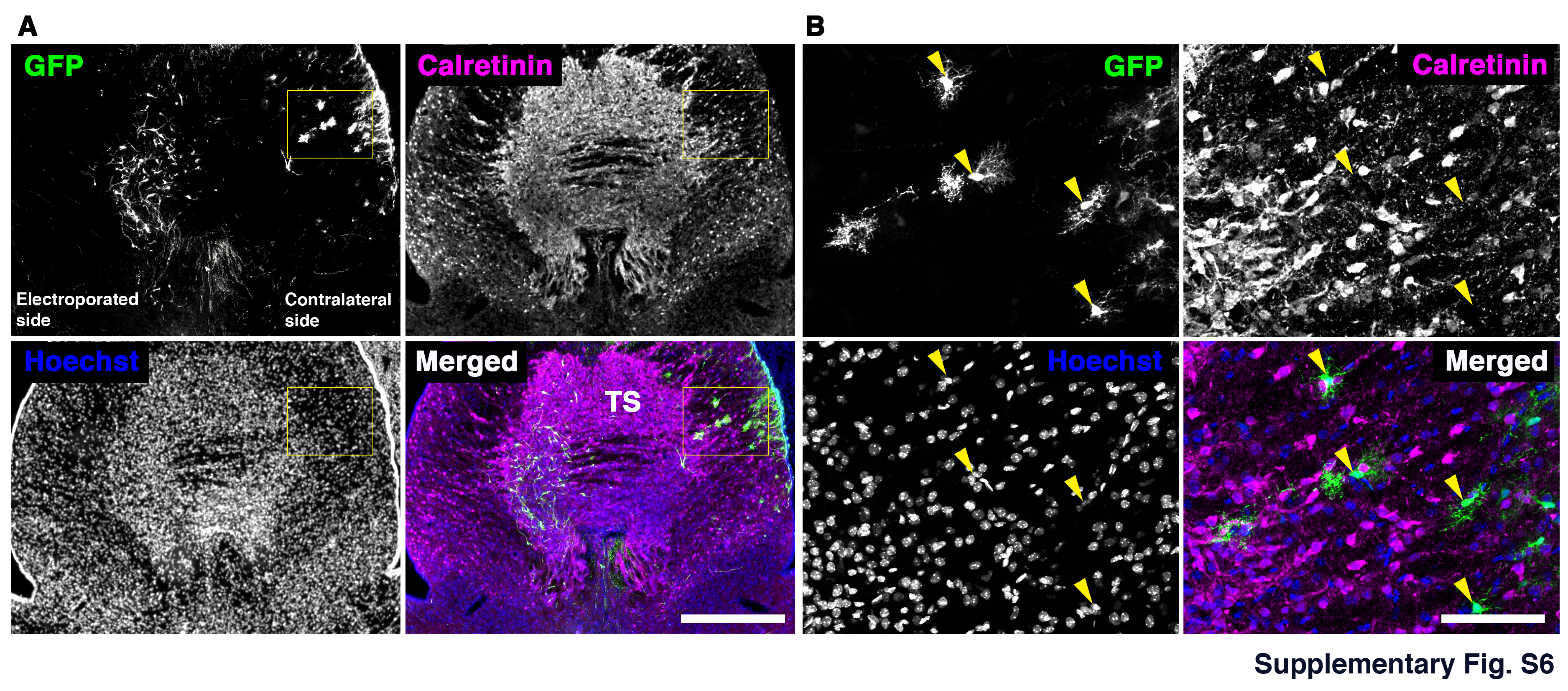


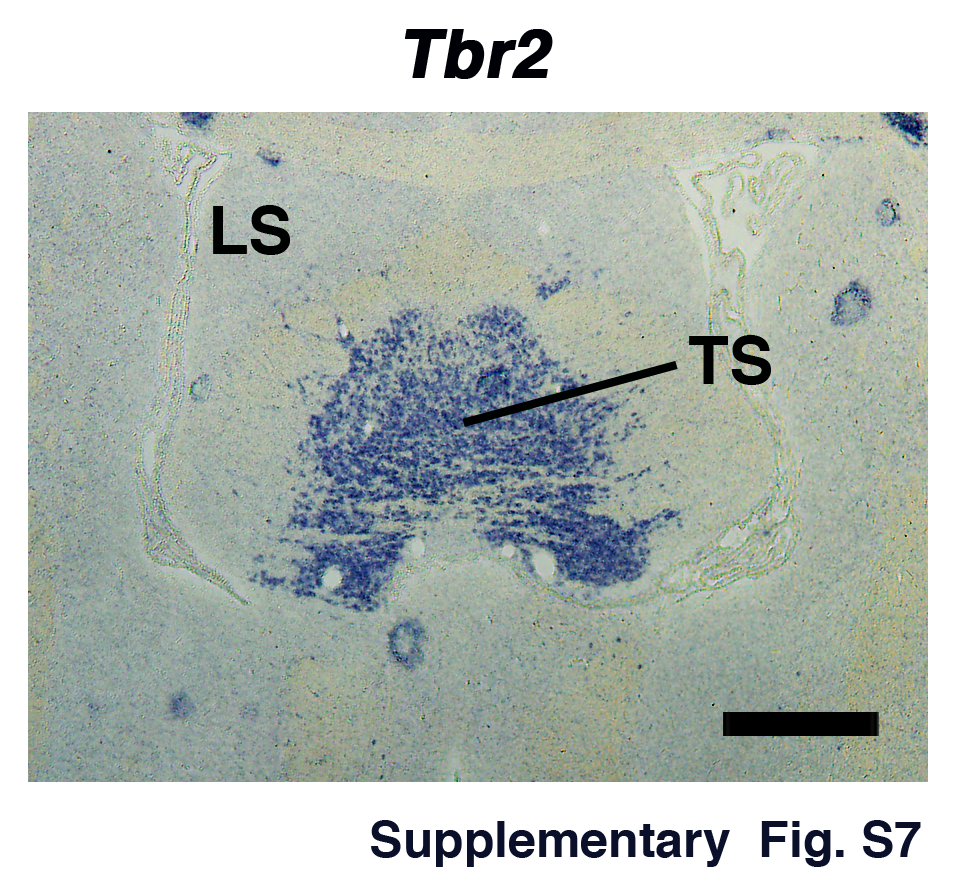


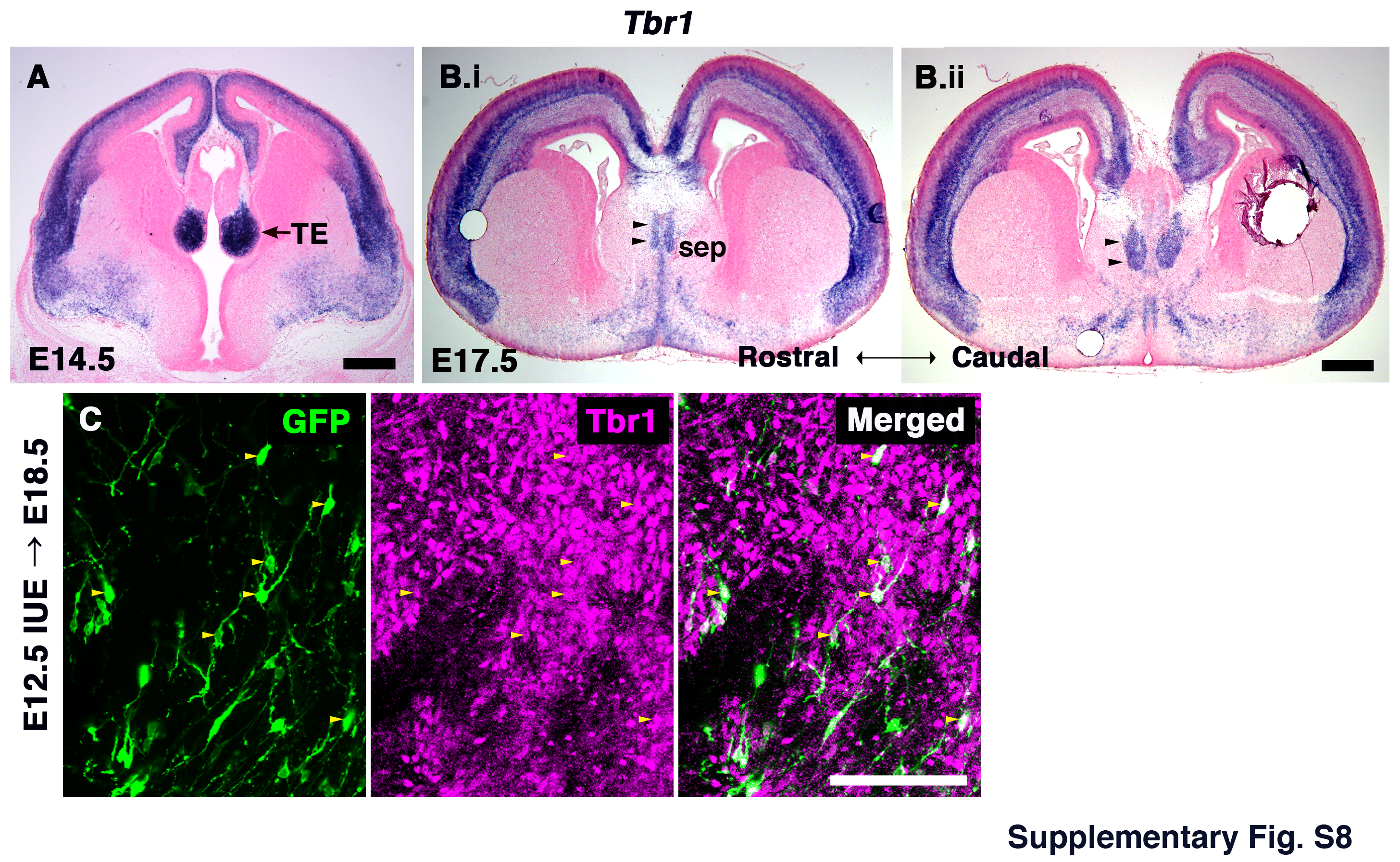


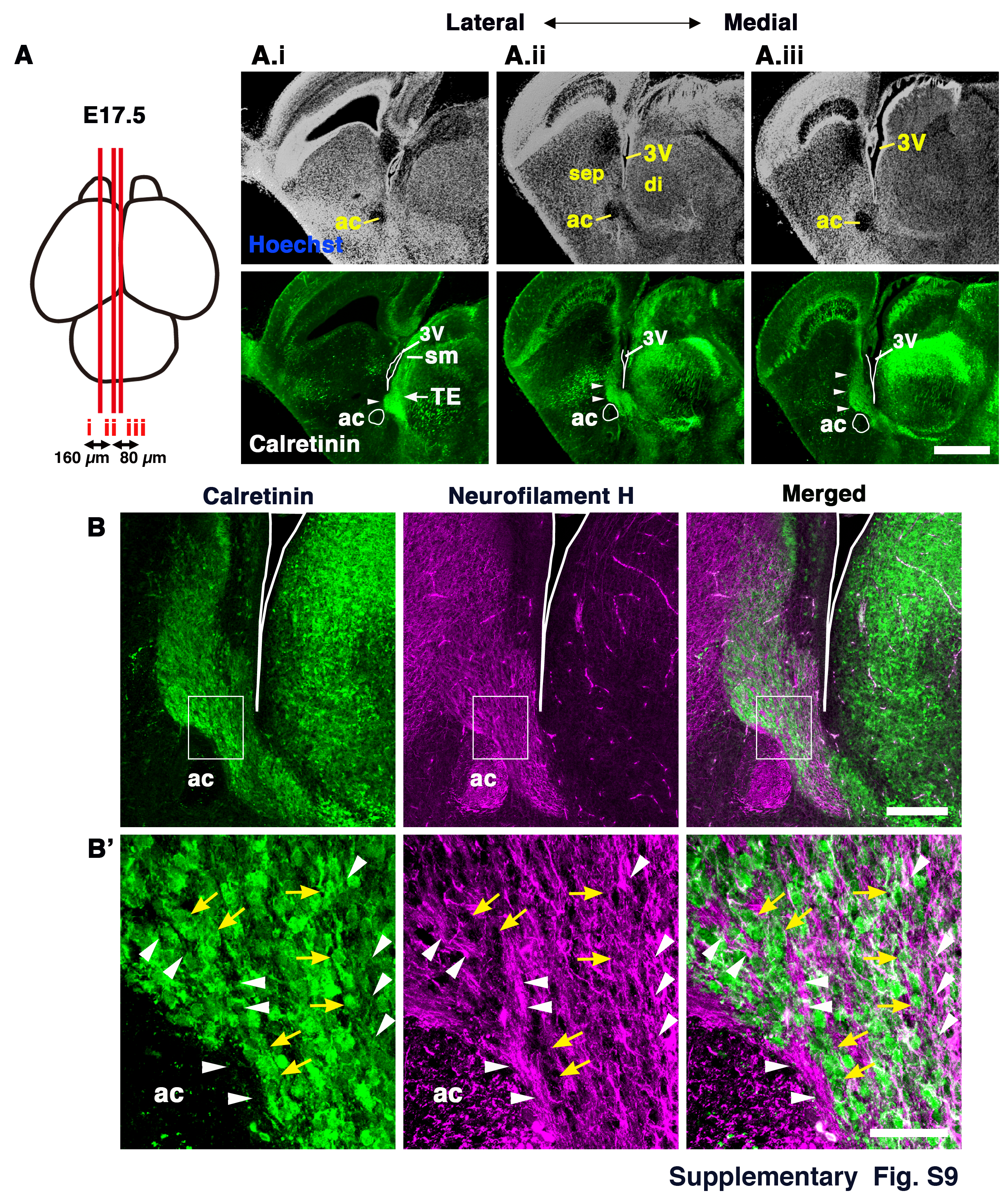

Supplement: Supplementary file 1 — supplementary information [file 41598_2018_30020_MOESM1_ESM.doc]
